# Effect of single-dose dexmedetomidine on postoperative recovery after ambulatory ureteroscopy and ureteric stenting: a double blind randomized controlled study

**DOI:** 10.1186/s12871-017-0464-6

**Published:** 2018-01-05

**Authors:** I. I. Shariffuddin, W. H. Teoh, S. Wahab, C. Y. Wang

**Affiliations:** 1 Department of Anaesthesia, Faculty of Medicine, University of Malaya, Lembah Pantai, 50603 Kuala Lumpur, Malaysia; 2 Wendy Teoh Pte. Ltd, Private Anaesthesia Practice, Singapore, Singapore

**Keywords:** Dexmedetomidine, Ureteroscopy, Minimum alveolar concentration, Pain, Postoperative, Sevoflurane, Recovery

## Abstract

**Background:**

Ambulatory surgery has recently gain popularity, as it is a good method of optimizinghospital resources utilization. To support ambulatory surgery, anaesthetic goals nowrevolve around patients’ early recovery with minimal pain and nausea, expedientdischarge home and prompt resumption of activities of daily living. In this study, weevaluated the effect of a single pre-induction dose of dexmedetomidine on anaestheticrequirements, postoperative pain and clinical recovery after ambulatory ureteroscopy andureteric stenting under general anaesthesia.

**Methods:**

Sixty patients were randomised to receive IV dexmedetomidine 0.5 μg.kg-^1^ (Group DEX, *n* = 30) or IV saline (Group P, *n* = 30). General anaesthesia was maintained with Sevoflurane: oxygen: air, titrated to BIS 40–60. Pain intensity, sedation, rescue analgesics, nausea/vomiting and resumption of daily activities were recorded at 1 h, and postoperative day (POD) 1–5.

**Results:**

Group DEX patients had significant reduction in sevoflurane minimum alveolar concentration (MAC), mean (SD) DEX vs. Placebo 0.6 (0.2) vs. 0.9 (0.1), *p* = 0.037; reduced postoperative resting pain at 1 h (VAS 0–10) (mean (SD) 1.00 (1.84) vs. 2.63 (2.78), *p* = 0.004), POD 1 (mean (SD) 1.50 (1.48) vs. 2.87 (2.72), *p* = 0.002), POD 2 (0.53 (0.97) vs. 1.73 (1.96), *p* = 0.001) and POD 3 (0.30 (0.75) vs. 0.89 (1.49), *p* = 0.001). DEX patients also had less pain on movement POD 1 (3.00 (2.12) vs. 4.30 (3.10), *p* = 0.043) and POD 2 (2.10 (1.98) vs. 3.10 (2.46), *p* = 0.040), with higher resumption of daily activities by 48 h compared to placebo, 87% vs. 63%, *p* = 0.04.

**Conclusions:**

We conclude that a single dose of dexmedetomidine was a useful adjuvant in reducing MAC and postoperative pain (at 1 h and POD 1–3), facilitating faster return to daily activities by 48 h.

**Trial registration:**

The Australian New Zealand Clinical Trials Registry (ANZCTR), ACTRN12617001120369, 31st July 2017, retrospectively registered.

## Background

There has been a paradigm shift in modern healthcare and hospital resource utilization, such that most surgical procedures are now performed in an ambulatory outpatient setting versus traditional hospitalization postoperatively [1]. Anaesthetic goals for ambulatory surgery revolve around patients’ early recovery with minimal pain and nausea [2], expedient discharge home and prompt resumption of activities of daily living. However, certain types of operations are associated with higher incidences of severe pain in the early recovery period. After ureteroscopic assisted ureteral stenting, significant flank and bladder pain can occur with reports of pain visual analogue scores (VAS) 6 upon 10 up to 48 h postoperatively [3–6]. Amelioration of this often necessitates increased parenteral or oral narcotic consumption with attendant side effects of somnolence, postoperative nausea/vomiting (PONV), clouded sensorium, respiratory depression and constipation [7] that are not only distressing but further delay discharge.

Dexmedetomidine is a highly selective α-2-agonist exhibiting dose-dependent sedation, analgesia, anti-inflammatory, sympatholytic and anxiolytic effects without relevant respiratory depression [8, 9]. The intraoperative use of dexmedetomidine has been reported to lower 1 h postoperative pain scores, reduce opioid consumption and result in a lower risk for opioid-related adverse events by some investigators [10]. However a recent Cochrane review of perioperative dexmedetomidine administration versus placebo in abdominal surgery for adults only managed to show opioid-sparing effects and failed to demonstrate conclusively important differences in postoperative pain scores [11]. The authors concluded that the clinical importance for patients remains uncertain, as the influence of dexmedetomidine on patient-important outcomes such as gastrointestinal function, mobilization and adverse effects could not be satisfactorily determined.

This clinical equipoise left questions unanswered for our institution as to whether it would benefit our patient cohort. We also found no studies evaluating the effect of a single adjuvant dose of dexmedetomidine on patients undergoing ureteroscopy in an ambulatory surgery setting and therefore embarked on this study. We aimed to evaluate the efficacy of a single pre-induction dose of Dexmedetomidine 0.5 μg.kg-^1^for ureteric stenting after ureteroscopy, and assess its impact on intraoperative anaesthetic agent requirements eg. minimum alveolar concentration (MAC) {primary outcome} and secondarily, postoperative pain scores, perioperative analgesia requirements, PONV, drowsiness and return to normal daily activities.

## Methods

The study was approved by our institutional review board (Medical Ethics Committee, University Malaya Medical Centre, ethics no: 672.7) and all patients gave their written informed consent. We enrolled patients of American Society Anaesthesiologists physical status I-II, aged between 18 and 65 years who underwent elective ureteroscopy and ureteric stenting in our ambulatory day care centre. We excluded patients with increased serum creatinine (> 200μmolL^−1^), advanced liver disease (liver enzymes twice the normal range or higher), those with a history of chronic use of sedatives/ narcotics or analgesics, known alcohol or drug abuse, allergy to study medication and those devoid of postoperative telephone access.

Patients were randomized into two groups, Group Dexmedetomedine (DEX) vs. Placebo using a computer generated random number table in blocks of ten. After recruitment, the enrolling investigators opened sealed opaque envelopes that concealed group allocation. Participants, trial investigators, attending anaesthetists providing general anaesthesia for the cases, and outcome assesors were blinded to group allocation.

As part of our routine practice, all patients received oral paracetamol 1 g when they entered the preoperative holding bay. Intravenous access was secured with a 20 Gauge catheter, and ECG, noninvasive arterial blood pressure, heart rate (HR), pulse oximetry and a Bispectral Index (BIS) forehead strip was applied. Haemodynamic variables were recorded at five-minute intervals but BIS monitoring (BIS monitor Model Aspect Medical Systems, Norwood, MA) was only commenced later in the operating room.

In this holding bay, patients randomised to Group DEX then received intravenous dexmedetomedine (Precedex™; Hospira Inc.,Lake Forest,IL,USA) 0.5 μg.kg^−1^ and the placebo group received 20mls of normal saline. The study drug was prepared by one of the investigators by drawing up the required dose and diluting it with normal saline to a total of 20 ml in a 20 ml syringe, which was then infused over 10 min via an infusion pump [Perfusor space, B Braun,USA] by a blinded nurse not involved in the trial. Patients in the placebo group received 20 ml normal saline in a similar syringe by the same method. The investigator that prepared the drug had no further role in assessing the patient’s outcomes.

Post-administration of the study drug, all patients were transferred to the operating rooms within 10 min, where the patients were positioned on the operating table with their heads resting on a jelly doughnut. Standard monitoring was applied and patients were preoxygenated with 100% oxygen for 3 min, before anaesthesia was induced with IV fentanyl 1 μg.kg^−1^ and propofol 2 mg.kg^−1^ or more until patient’s exhibited loss of verbal response. A classic laryngeal mask airway size 3 or 4 was then inserted when the jaw was sufficiently slack. Effective ventilation was confirmed on capnography and anaesthesia maintained with Sevoflurane in an oxygen: air mixture. Depth of anaesthesia was titrated to maintain BIS values between 40 to 60. Intraoperative IV fentanyl in 50 μg boluses were given if deemed necessary by the attending anaesthetist managing the case. No routine anti-emetics were administered. The blood pressure, HR, mean alveolar concentration (MAC) of Sevoflurane and BIS values were recorded every 5 min. Upon completion of surgery, the LMA was removed in the operating room when patients were awake and obeying commands.

Postoperatively, patients were assessed in the Post Anaesthesia Care Unit (PACU) within the hour for sedation levels (using Ramsay Sedation Score [12] of 1 = anxious, agitated or restless; 2 = cooperative, orientated, tranquil; 3 = responds to commands; 4 = asleep but brisk response to light glabellar tap or loud auditory stimulus; 5 = asleep, sluggish response to light glabellar tap or loud auditory stimulus; 6 = asleep, no response), pain intensity (on Visual Analogue Score, VAS 0–10), incidence of nausea and vomiting, need for rescue analgesia (IV fentanyl 25 μg boluses) and anti-emetics (IV metoclopramide 10 mg). Patients were deemed fit for discharge when they were haemodynamically stable, had tolerated a snack (chocolate drink and biscuits) without undue nausea/vomiting, able to stand, void and ambulate with minimal assistance. All patients were discharged home with 5 days worth of oral Celebrex 200 mg b.d.

Two research nurses who were blinded to patient group allocation then conducted a standardised phone interview with the patients daily between 1700 and 1900 h on postoperative day (POD) 1–5. Patients were quizzed on their pain at rest (during normal tidal volume breathing) and movement (rising from supine position), incidence of nausea/ vomiting and their ability to ambulate and resume daily activities.

### Statistical analysis

The sample size was based on our primary outcome measure of anaesthetic minimum alveolar concentration (MAC). From an initial pilot study involving 16 patients, we found the mean (SD) MAC of Sevoflurane to be 0.85 (0.19). We deemed a MAC reduction of 0.25 to be a clinically significant difference between the two groups. Prospective power analysis indicated that with α = 0.05 and power of 80%, 25 patients per group were needed. Therefore we recruited 30 patients per group to account for dropouts. Parametric data and non-parametric data was analysed with Student’s t test and Mann-Whitney U-test respectively, and Fisher’s exact test was used to compare side effects using SPSS 15.0 ™ (SPSS Inc., Chicago, IL, USA) software. A *p* value of <0.05 was deemed statistically significant.

## Results

**Fig. 1:**
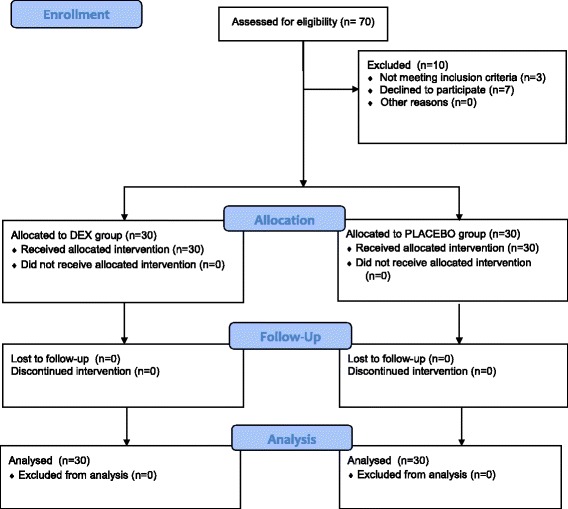
Flow of patients through the study

Seventy patients were recruited to the study (Fig. 1) and sixty were eventually randomised after fulfilling eligibility criteria. Baseline demographics of the patients were comparable, barring an older age group in the placebo group occurring by chance (Table 1).

**Table 1:** Baseline characteristics of patients receiving intravenous dexmedetomidine 0.5 μg.kg^−1^ or placebo. Values are mean (SD) and numbers of patients (n)

	Dex (*n* = 30)	Placebo (*n* = 30)
Age; years	39.2 (11.2)	46.3 (13.1)
Weight; kg	66.6 (9.4)	65.8 (13.1)
ASA class (I: II)	26/4	19/11
Gender (M: F)	25:5	22:8
Duration of surgery; min	50.7 (22.3)	45.5 (15.7)
Induction dose of propofol; mg	137.7 (22.3)	145.6 (35.6)
Intraoperative fentanyl; μg	20.9 (21.7)	26.7 (18.5)
Ureteroscopy & ureteric stenting		
single	27	28
bilateral	3	2

**Fig. 2:**
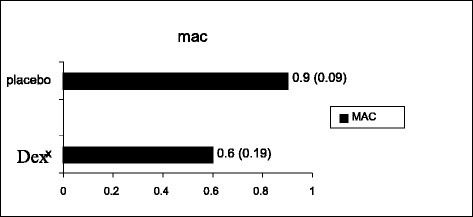
Mean (SD) minimum alveolar concentration (MAC) of patients who received dexmedetomidine compared to placebo. Minimum Alveolar Concentration (MAC)

**Fig. 3:**
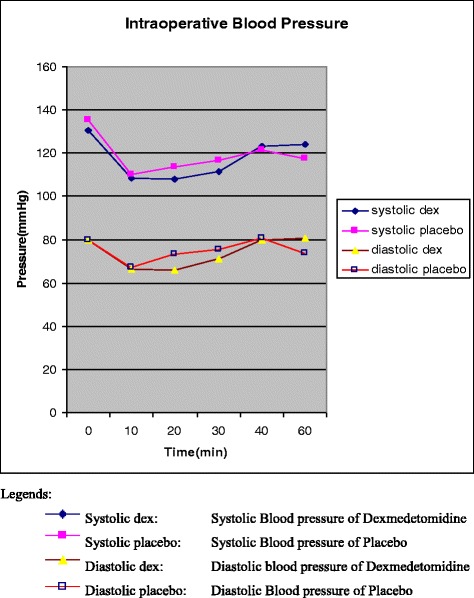
Intraoperative haemodynamic changes

Intraoperatively, we found a significant reduction in the mean (SD) MAC of the anaesthetic agent in patients who had received dexmedetomidine compared to placebo, 0.6 (0.2) vs. 0.9 (0.1), *p* = 0.037 (Fig. 2). There were no differences in haemodynamics between the two groups (recorded from the time the infusion commenced and continued intraoperatively) except at 15 min, where there was a significant lowering of the mean (SD) DEX systolic blood pressure 104.3 (12.8) vs.114.2 (21.2) mmHg, diastolic blood pressure 62.3 (11.8) vs.72.2 (19.2) mmHg, and heart rate 62.6 (10.5) vs. 69.7 (12.1) compared to placebo (all *p* < 0.05) which then became insignificant at the 20 min mark (Fig. 3).

Intraoperatively both groups received the same amount of fentanyl but the mean (SD) VAS for postoperative pain 1 h after PACU arrival was significantly reduced in the dexmedetomidine group, 1.00 (1.84) vs. 2.63 (2.78), *p* = 0.004. Correspondingly, no patient in the DEX group required rescue fentanyl in the PACU, whereas the placebo group received a median [range] of 0 [0–100] μg, *p* = 0.040 (Table 2). There was no difference in the incidence of nausea/ vomiting or use of anti-emetics. Median [range] sedation score for both DEX and placebo were comparable, 2 [2–4] vs. 2 [2–3], *p* = 0.160.

**Table 2:** Post-anaesthesia care unit (PACU) recovery profile of patients who received intravenous dexmedetomidine 0.5 μg.kg^−1^ or placebo. Values are mean (SD), median [range], numbers of patients (n) or proportions (%)

	Dex (*n* = 30)	Placebo (*n* = 30)	*P* Value
PACU fentanyl; μg	0	0 [0–100]	0.040*
PACU anti-emetics; Yes / No	0: 30	4: 26	0.110
Ramsay Sedation Score	2 [2–4]	2 [2–3]	0.160
Nausea and vomiting	0 / 30 (0%)	2 /30 (6.7%)	0.492
Unexpected admission	0 / 30 (0%)	2 / 30 (6.7%)	0.492

*Indicates statistically significant *p* value

Two patients in the placebo group required unexpected hospital admission. One patient had uncontrolled pain postoperatively (VAS 10/10) despite receiving IV Tramadol 100 mg in addition to fentanyl rescue in the PACU and requested admission for pain control and rest for 3 days. Another patient had persistent haematuria and was admitted for observation overnight on a suspicion of sepsis but his haemoglobin level was 12.8 g/dL, platelets 271 × 10^9^; he remained afebrile with a normal total white count and was discharged the next day.

**Fig. 4:**
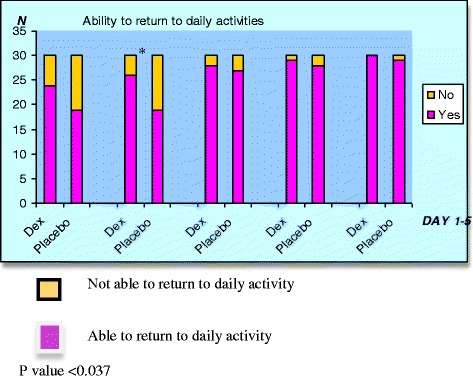
Number of patients who were able to resume their daily activities on postoperative Day 1–5

Upon discharge home, patients that had received DEX had significantly reduced pain scores at rest on the first to third postoperative day, and reduced pain on movement on POD 1 and POD 2 compared to placebo [Table 3]. Significantly more DEX patients resumed their normal daily activities on the 2nd postoperative day than placebo, 26/30 (87%) vs. 19/30 (63%), *p* = 0.037 [Fig. 4]. On POD 4 patients who received DEX continued to exhibit lower pain scores at rest and upon movement although this did not achieve statistical significance, and by POD5, this advantage was only seen on movement (Table 3).

**Table 3:** Pain scores at rest and upon movement in the Post-Anaesthesia Care Unit (PACU) and on postoperative day (POD) 1–5. Values are mean (SD)

Drugs	Dex (*n* = 30)	Placebo (*n* = 30)	*P* Value
PACU	1.00 (1.84)	2.63 (2.78)	0.004*
POD1			
Rest	1.50 (1.48)	2.87(2.72)	0.002*
Movement	3.00 (2.12)	4.30(3.10)	0.043*
POD2			
Rest	0.53(0.97)	1.73(1.96)	0.001*
Movement	2.10(1.98)	3.10(2.46)	0.040*
POD3			
Rest	0.30(0.75)	0.89(1.49)	0.001*
Movement	1.60(1.73)	2.10(1.98)	0.630
POD4			
Rest	0.17(0.64)	0.29(0.71)	0.276
Movement	0.80(1.56)	1.00(1.49)	0.987
POD5			
Rest	0.07(0.36)	0.07(0.37)	0.923
Movement	0.30(1.95)	0.77(1.40)	0.308

*Indicates statistically significant *p* value

## Discussion

In this study, we demonstrated that a single pre-induction dose of dexmedetomidine 0.5 μg.kg^−1^ administered to patients undergoing ureteroscopy and ureteric stenting, not only significantly reduced the MAC of sevoflurane and intraoperative anaesthetic requirements, but also resulted in a significant 60% reduction in immediate postoperative pain that extended to Day 3 post discharge. This was highly clinically significant allowing patients earlier resumption of their daily activities and return to the workforce without being hindered by pain occurring after discharge that would negatively impact patient recovery and place economic burdens on patient and caregiver [13].

α^2^-Adrenergic agonists produce clinical effects after binding to α^2^-adrenergic receptors, of which there are three subtypes (α^2A^, α^2B^, and α^2C^). These receptor subtypes are distributed ubiquitously, and early mice studies investigating substitution of a mutant α^2A^-adrenergic receptor via ‘hit and run’ gene targeting revealed the role of this subtype in sedative, analgesic, and anesthetic-sparing responses in vivo [14]. In this aspect, our study echoes the findings of other investigations in humans that use minimum alveolar concentration (MAC) as a measure of anesthetic potency. Others have demonstrated that women undergoing abdominal hysterectomy who received dexmedetomidine infusions (target plasma concentration of 0.6 ng.ml^−1^) had a 47% reduction in the MAC of isoflurane, compared to without dexmedetomidine [15]. Similarly an infusion of dexmedetomidine 0.7 ng.ml^−1^ administered 15 min pre- induction of anaesthesia also decreased the MAC of sevoflurane by 17% in adults aged 55–70 years [16]. Other investigators who used BIS monitoring 40–60 to titrate their end-tidal Sevoflurane concentrations also found a reduction in MAC in surgical patients receiving continuous dexmedetomidine infusions at 0.5 μg.kg.hr^−1^ with relative haemodynamic stability [17]. We found this reduction in MAC with just a small single dose of dexmedetomidine administered preoperatively, echoing the findings of Lawrence CJ et al. who found that a single pre-induction intravenous dose of dexmedetomidine 2 μg.kg^−1^ resulted in lower mean intraoperative isoflurane concentration in the dexmedetomidine-treated patients than controls undergoing minor orthopaedic and general surgery [18].

The analgesic properties of dexmedetomidine are conferred by its central action at the locus coeruleus in the brainstem and spinal cord that inhibits neuronal firing, thereby triggering sedation, anxiolysis and analgesia [19]. We found that a single pre-induction dose of dexmedetomidine 0.5 μg.kg^−1^ reduced immediate postoperative pain by a dramatic 60% in our study. This resonates with Schnabel’s [10] meta-analysis of 28 randomised controlled trials involving 1420 adults that also reported lower postoperative pain at 1 h (but unlike them we did not find any opiod sparing effects or significant intraoperative bradycardia). Further analyzing 11 randomized controlled trials, 434 children receiving dexmedetomidine demonstrated a reduced relative risk for postoperative pain in comparison with placebo, although the influence of dexmedetomidine on postoperative opioid consumption was less clear in this meta-analysis [20].

On the other hand, other investigators have found no difference in immediate postoperative pain but a reduced amount of rescue analgesics after a single dose of dexmedetomidine [21]. A recent Cochrane review [11] that included 402 adults undergoing abdominal surgery who received dexmedetomidine found a reduction in ‘rescue’ opioid consumption, but no clinically perceptible differences in postoperative pain (visual analogue scale (VAS) 0 to 100 mm, where 0 = no pain and 100 = worst imaginable pain) in the first 24 h after surgery when compared with placebo. However the authors opined that the quality of the evidence was very low as the result of imprecision, methodological limitations and substantial heterogeneity among the six included studies.

We demonstrated that a single pre-induction dose of dexmedetomidine 0.5 μg.kg^−1^ resulted in significantly reduced pain at rest for the first three postoperative days, and pain on movement on postoperative day 1 and 2. More importantly, our study significantly showed that this extended analgesic duration actually enhanced the recovery profile and enabled a significantly higher proportion of patients to return to their daily activities after 48 h as compared to the placebo group. In a rat model of incisional pain, pretreatment with dexmedetomidine (administered subcutaneously 30 min before plantar incision) significantly decreased remifentanil-induced postoperative hyperalgesia as indicated by increased paw withdrawal latencies and thresholds to thermal and mechanical stimulation [22]. Western blotting experiments revealed that the antihyperalgesic effects of dexmedetomidine were associated with suppression of spinal cord N-methyl-d-aspartate receptor (NMDAR) excitability, as measured by a reduction in NR2B (NMDR receptor 2B subunit) tyrosine phosphorylation (Tyr1472 site), which was upregulated after remifentanil infusion [22]. These results correlated with the rat’s pain behavior changes suggesting that dexmedetomidine could efficiently alleviate opiod-induced hyperalgesia and we postulate this to be the underlying reason for its extended analgesic benefits beyond its half-life, even though administered as a single dose.

Furthermore, surgical injury to tissue results in increased stress hormone production (C-reactive protein, cortisol, catecholamines), complement system activation, leukocyte migration to the site of injury, and release of cytokines (interleukins, tumor necrosis factor), superoxide radicals, proteases and growth factors [23]. Although an appropriate inflammatory cascade is essential for tissue reconstitution, restoration of homeostasis and infection control, these inflammatory mediators may induce fatigue and prolong convalescence in otherwise healthy patients. The choice of anaesthetic technique can affect both immunostimulatory and immunosuppressive mechanisms; directly by modulating immune cell function or indirectly by attenuating the stress response thereby impacting short and long-term patient outcomes.

There is already evidence that dexmedetomidine decreases production of inflammatory cytokines while lowering intra-abdominal pressure in critically ill patients with sepsis [9]. In animals, dexmedetomidine attenuates the increase of plasma cytokine levels after endotoxin injection and drastically reduces the mortality rate of infected animals [24]. These results lend weight to the role of dexmedetomidine in preventing untoward stress responses, possibly mitigating delays in post-surgical convalescence and enhancing quality of recovery from surgery. Indeed, Bekker et al. found that patients who were administered a dexmedetomidine infusion during multilevel spinal fusions exhibited an improved quality of recovery (global 40-item quality of recovery questionnaire scores, QoR40) and reduced fatigue (assessed using Fatigue Severity Scores, FSS) on the third postoperative day, with reduced plasma cortisol and interleukin10 levels in comparison with the control group [25]. Using the same QoR40 and FSS benchmarks, Ge et al. also found that abdominal hysterectomy patients who received a propofol/ remifentanil/ dexmedetomidine anaesthetic showed higher recovery scores from the third postoperative day [26]. These data support our own study’s and explains how a single dose of dexmedetomidine could confer far-reaching extended benefits to our patient cohort in terms of reduced postoperative pain for 3 days and faster return to their regular lifestyles.

Our study did have the following limitations: preoperative assessment could have ideally screened for motion sickness and smoking as possible confounders of the risk of PONV. We did not measure plasma catecholamine or stress-hormone concentrations; although these measurements could support a direct relationship between sympatholytic and anti-inflammatory properties of dexmedetomidine and postoperative quality of recovery, it was not logistically feasible under the constraints of our study setting. Additionally as MAC decreases 4% to 5% per degree centigrade decrease in core temperature [15], temperature monitoring could have been applied. However we did not do this as surgery was relatively short averaging 45–60 min, and we had applied a forced air warmer to the upper bodies of all patients to maintain normothermia.

## Conclusions

In conclusion, we found that the administration of a single pre-induction dose of dexmedetomidine 0.5 μg.kg^−1^ reduced intraoperative anaesthetic agent requirements, alleviated immediate postoperative pain in hospital and up to the third postoperative day at home, enabling patients earlier resumption of their daily activities. We conclude that dexmedetomidine proved to be a useful adjuvant that benefitted our patients undergoing ureteroscopy and ureteric stenting in our ambulatory clinical practice.

## Abbreviations

BIS: Bispectral Index
DEX: Dexmedetomedine
FSS: Fatigue Severity Scores
HR: Heart rate
MAC: Minimum alveolar concentration
PONV: Postoperative nausea/vomiting
QoR40: global 40-item quality of recovery questionnaire scores
VAS: Visual analogue scores
